# *Capparis spinosa* Alleviates DSS-Induced Ulcerative Colitis via Regulation of the Gut Microbiota and Oxidative Stress

**DOI:** 10.1155/2021/1227876

**Published:** 2021-12-15

**Authors:** Xiaoting Zhu, Yi Yang, Weizhen Gao, Bingjie Jiang, Lei Shi

**Affiliations:** College of Life Sciences, Xinjiang Agricultural University, Urumqi 830052, Xinjiang, China

## Abstract

Ulcerative colitis (UC) is a chronic inflammatory disease. Here, the potential effects of *Capparis spinosa* water extract (CSWE) on colonic histopathology, inflammation, and gut microbiota composition in dextran sulfate sodium (DSS) induced UC mice were evaluated. Our results showed that CSWE treatment improved the colonic histopathology of UC mice, increased the levels of tight junction protein gene *ZO-*1 and *Occludin* in intestinal epithelial cells, and inhibited the expression of proinflammatory cytokines (*IL-1β*, *IL-*6, and *TNF-α*). Furthermore, CSWE administration alleviated oxidative stress in the colon of UC mice. The effects of CSWE on the compositions and metabolomic profiles of the gut microbiota in UC mice were investigated. It was found that CSWE could enhance the diversity of gut microbes and the abundance of probiotics and metabonomics had the strongest association with Firmicutes. Our results indicated that CSWE might be an ideal candidate as a potential therapeutic natural product for the treatment of UC.

## 1. Introduction

Inflammatory bowel disease (IBD), including ulcerative colitis (UC) and Crohn's disease, is an idiopathic, chronic, and recurrent disease [1]. UC is a chronic inflammatory disease characterized by mucosal inflammation of the colon and rectum, with typical symptoms of rectal bleeding, diarrhea, and urgency [2]. The highest prevalence of UC has been reported in Europe at 505 cases per 100,000 people (approximately 0.5%) [3]. Due to changes in lifestyles and eating habits, the prevalence rate of UC in Asia has been increasing every year [4]. It has been reported that UC can be triggered and proceed through the interaction of genetic factors, environmental factors such as stress and food habits, excessive production of inflammation-related cytokines, and abnormal immune responses in the mucosal and submucosal layers of the intestine [5]. A number of drug classes are available for the treatment of UC, including salicylates, corticosteroids, thiopurines, calcineurin inhibitors, antitumor necrosis factor (TNF) agents, and antiadhesion molecules [6]. Despite the availability of various therapeutic agents for the treatment of UC, routine treatment has limitations and serious side effects, such as drug dependence, disruption of the immune system, and irreversible complications, including hypertension and gastric ulcer [7]. Therefore, a new drug that safely and effectively treats UC is urgently needed.

Biological macromolecules exist in traditional Chinese medicinal plants and edible mushrooms and are well known to have multiple physiological functions [8]. Polysaccharides from natural resources are complex carbohydrates that are unable to be hydrolyzed by digestive enzymes in the human intestine but are metabolized by gut microbiota to produce short-chain fatty acids (SCFAs), which have recently become a research hotspot due to their therapeutic effects on UC [9, 10]. It was shown that *Scutellaria baicalensis* Georgi polysaccharides ameliorate dextran sulfate sodium (DSS) induced UC by improving intestinal barrier function and modulating gut microbiota [11]. *Ficus carica* polysaccharides (FCPSs) alleviated colitis by improving colon length and suppressing the infiltration of inflammatory cells in the gut, indicating that the protective effects of FCPSs on UC might be highly correlated with microbiota compositional changes and the formation of SCFAs [12].

*Capparis spinosa* belongs to the Capparaceae family, which is mainly distributed in the Xinjiang Uygur Autonomous Region in China, and its edible parts are commonly known as capers [13, 14]. Accumulating evidence has shown that *C. spinosa* contains many biologically active chemical groups, including polysaccharides, alkaloids, glycosides, tannins, phenolic compounds, and flavonoids, which exert various pharmacological functions [15], such as antioxidant [16], anti-inflammation [17], antitumor [18], antiarthritic [19], antidiabetic [20], and immunomodulatory [21] activities. However, the effect of *C. spinosa* on UC, a representative inflammatory disease, has not yet been identified.

In this study, *C. spinosa* water extract (CSWE) was prepared, and the polysaccharides are the main component (37.6%). Here, we aimed to elucidate the protective effects of *C. spinosa* water extract (CSWE) on UC and further explore the relevant mechanisms. The effects of CSWE on the body weight, colon length, degrees of colon lesions, colonic antioxidant system, inflammatory cytokine levels, and gut microbiota constitution in DSS-induced UC mice were investigated. Our study demonstrated that CSWE possessed potential health benefits on UC.

## 2. Materials and Methods

### 2.1. Preparation of CSWE

One hundred grams of *C. spinosa* fruit was collected from Turpan City in Xinjiang Uygur Autonomous Region, China. CSWE was prepared according to the water extraction and alcohol precipitation method. Cleaned *C. spinosa* fruit was ground to a fine powder, which was extracted three times with 1000 ml of distilled water with stirring at 80°C for 3 h, followed by 30 min of sonication at 300 W and 50°C. The extracts were pooled together and centrifuged at 3000 rpm for 15 min. Distilled water was subsequently removed from the extract using a rotary vacuum evaporator at 55°C. The extract was deproteinized by the Sevage method and precipitated with 4 volumes of ethanol at 4°C overnight. After centrifugation at 8000 rpm for 15 min, the extract was collected, and the remaining solvent was removed by air drying at room temperature to obtain the water extract. Extracts were reconstituted in distilled water and sterilized with a 0.22 mm filter. The content of polysaccharides measured by the phenol-sulfuric acid method was 37.6%. The content of protein was 3.5%.

### 2.2. Animals and Ethics Statement

Kunming male mice (6–8 weeks age) were purchased from the Animal Laboratory Center, Xinjiang Medical University (Urumqi, Xinjiang, China), and housed in a temperature-controlled, light-cycled animal facility of Xinjiang Agricultural University. Animal experimental procedures followed the National Institutes of Health guidelines for the care and use of laboratory animals. All experimental procedures involving animals were approved (animal protocol number: 2020022) by the Animal Welfare and Ethics Committee of Xinjiang Agricultural University, Urumqi, Xinjiang, China.

### 2.3. In Vivo Study

The mice were randomly assigned into five groups (*n* = 7), including an untreated group, DSS group, CSWE(L) group, CSWE(H) group, and a 5-ASA positive control group. All mice were given a standard diet (Medicience, Jiangsu, China) and drink freely. The control group was given distilled water, while the other groups were given 3% (wt/vol) DSS (MW 50000) for 7 days to induce the UC mouse model. After DSS administration, the CSWE(L) group and CSWE(H) group were gavaged with 200 and 400 mg/kg/day CSWE, respectively, and the 5-ASA group was gavaged with 220 mg/kg/day mesalamine for 7 days. The body weight and stool occult blood degree were recorded every other day during the experimental period, and the severity of UC was assessed daily using a disease activity index (DAI).

### 2.4. Histopathological Analysis of the Colon and Small Intestinal Tissue

Colon and small intestinal segments were fixed in 4% polyformaldehyde stationary solution for 24 h. After dehydration, the sections were embedded in paraffin and then sectioned to 4 *μ*m thickness. Sections were stained with hematoxylin and eosin (HE). The histopathologic features of the colon and small intestinal tissue sections were evaluated by light microscopy (Olympus, Tokyo, Japan). The histological scores were calculated according to the damages of inflammatory infiltration and epithelium mucosa [22]. The standard for pathological scoring is shown in Table 1. Furthermore, goblet cells were stained using the Glycogen (PAS) Stain Kit (Jiancheng, Nanjing, China).

### 2.5. qRT-PCR Analysis

RNA was extracted from colon tissues using TRIzol reagent (Solarbio, Beijing, China) according to the manufacturer's protocol. First-strand cDNA was synthesized using a reverse transcription kit (Foregene, Chengdu, China) according to the manufacturer's instructions. qRT-PCR was performed with Easy^TM^-SYBR Green Master Mix (Foregene, Chengdu, China) using a CFX96 real-time PCR machine (Bio-Rad). PCR was performed under the following conditions: 95°C for 10 min, 40 cycles of 15 s at 95°C, 30 s at 52–60°C (based on the target), and 45 s at 72°C. Data were normalized to *GAPDH*, and the relative expression levels of the genes were calculated using the 2^−ΔΔCt^ method. Detailed primer sequences are shown in Table 2.

### 2.6. Antioxidant Enzyme Activity Determination

The colon tissues were cut into small pieces of equal weight and homogenized on ice. The superoxide dismutase (SOD) and catalase (CAT) activities and total antioxidant capacity (T-AOC) and malondialdehyde (MDA) contents were measured using a Foregene (Chengdu, China) assay kit in accordance with the manufacturer's instructions.

### 2.7. Immunohistochemistry

Occludin and NF-*κ*B P65 (Bioswamp, Wuhan, China) were used as primary antibodies for immunohistochemical staining. Sections were incubated with 3% H_2_O_2_ for 10 min to block endogenous peroxidase activity and washed with PBS three times. Then, a 10% goat serum blocking solution was added for 10 min to block excessive protein. The sections were incubated with primary antibodies (1 : 100) overnight. After washing three times with PBS, the sections were incubated with conjugated HRP (1 : 1000) for 10 min. Then, coloration was conducted using a 3,3′-diaminobenzidine tetrahydrochloride hydrate (DAB) chromogenic reagent.

### 2.8. Fecal 16S rRNA Analysis

The mixed feces of mice in each group were collected at the end of this study (on day 15) and stored at −80°C after flash-frozen in liquid nitrogen. The DNA of total bacteria in mouse feces was extracted, then the V3-V4 hypervariable regions of the microbiota 16S rRNA gene were amplified by PCR using a primers 338F (5′-ACTCCTACGGGAGGCAGCA-3′) and 806R (5′-GGACTACHVGGGTWTCTAAT-3′), and the products were purified and quantified. The qualified products were directly sequenced with an Illumina HiSeq 2500 at Biomarker Technologies Co., Ltd. (Beijing, China).

### 2.9. Metabolomic Analyses

The LC-MS system utilized for metabolomic analysis was composed of a Waters Acquity I-Class PLUS ultraperformance liquid chromatography system coupled to a Waters Xevo G2-XS QTof high-resolution mass spectrometer. The chromatographic column used was an Acquity UPLC HSS T3 column (1.8 *µ*m, 2.1 mm × 100 mm) purchased from Waters. The mobile phase consisted of aqueous formic acid solution (A) and acetonitrile (B), and the gradient elution program was as follows: 0 min, 98%; 0.25 min, 98%; 10 min, 2%; 13 min, 2%; 13.1 min, 98%; 15 min, 98% and 0 min, 2%; 0.25 min, 2%; 10 min, 98%; 13 min, 98%; 13.1 min, 2%; and 15 min, 2%. The Xevo G2-XS QTof high-resolution mass spectrometer can collect primary and secondary mass spectral data in MSE mode under the control of acquisition software (MassLynx V4.2, Waters). In each data acquisition cycle, dual-channel data acquisition with low collision energy and high collision energy can be carried out simultaneously. The low collision energy was 2 V, the high collision energy was 10–40 V, and the scanning frequency was 0.2 s. The parameters of the electrospray ionization (ESI) source were as follows: capillary voltage, 2000 V (positive ion mode) or −1500 V (negative ion mode); taper hole voltage, 30 V; ion source temperature, 150°C; desolvation gas temperature, 500°C; back blowing flow rate, 50 L/h; and flow rate of desolvation gas, 800 L/h.

### 2.10. Statistical Analysis

The results were analyzed by using GraphPad Prism software 5.0 and expressed as the mean ± standard error of the mean (SEM) of at least triplicate experiments. Statistical significance was identified by using a one-way analysis of variance (ANOVA). QIIME software was used to generate species richness tables at different taxonomic levels, and then, the R language tool was used to draw the community structure map of samples at each taxonomic level. PICRUSt2 software was used to annotate the predicted characteristic sequence with the existing phylogenetic tree in the program, and IMG microbial genomic data were used to output functional information and infer the composition of functional genes in the sample. ClusterProfiler was used to enrich and analyze the annotation results of the Kyoto Encyclopedia of Genes and Genomes (KEGG) pathways and draw the classification map, and heat maps were generated using a hierarchical clustering algorithm to visualize the metabolite difference within the data set. The association analysis between the gut microflora-related metabolites and gut bacterial species was generated using Pearson's correlation coefficient. Values of *P* < 0.05 were considered to be statistically significant.

## 3. Results

### 3.1. CSWE Alleviates Colitis Symptoms in DSS-Induced UC Mice

**Figure 1:**
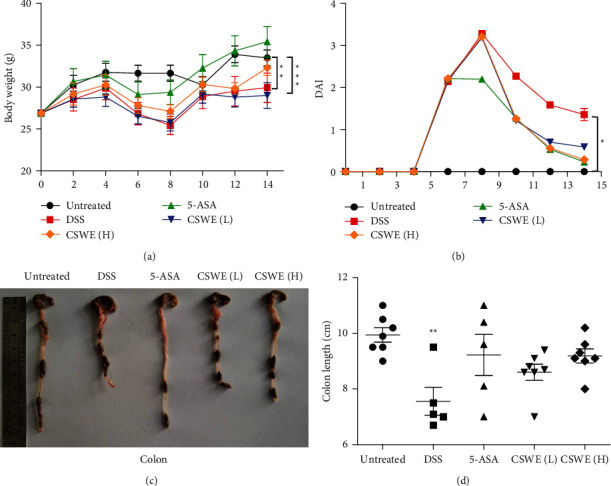
Effects of CSWE in DSS-induced UC in mice. (a) Body weight. (b) DAI score. (c) Photographs of the mouse colon in each group. (d) The colon length of mice in each group. All values are presented as the mean ± SEM. ^*∗*^*P* < 0.05, ^*∗∗*^*P* < 0.01, and ^*∗∗∗*^*P* < 0.001 compared to the untreated group.

As shown in Figure 1(a), mice in the DSS and CSWE(L) groups showed significant body weight loss compared with that in the untreated group (*P* < 0.01). However, CSWE(H) and 5-ASA treatment attenuated body weight loss in UC mice. As a result, the DAI score, which assesses UC severity, increased significantly after DSS treatment, whereas it was distinctly lower after CSWE and mesalazine treatment (Figure 1(b)). Furthermore, the colon length was significantly shorter in the mice in the DSS group than in mice in the other groups, while the colonic shortening induced by DSS was restored in a dose-dependent manner by CSWE (Figures 1(c) and 1(d)).

### 3.2. CSWE Decreases Histological Changes in the Colon of DSS-Induced UC Mice

**Figure 2:**
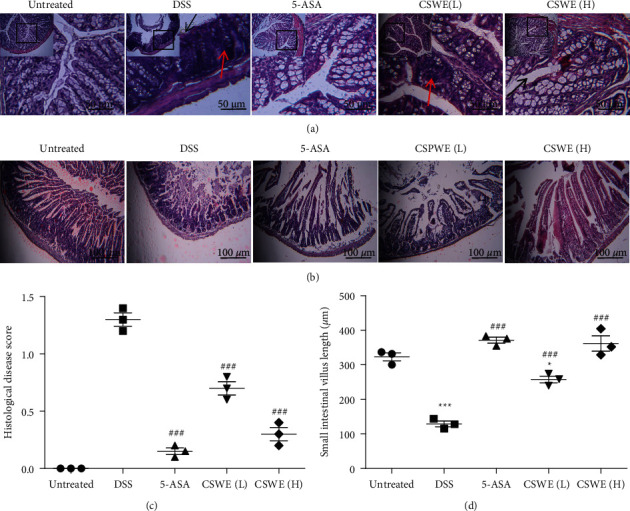
Effect of CSWE on colon pathological changes in DSS-induced UC mice. (a) Representative histological photos of the colon in each group (×100 and ×200). The black arrow indicates crypts, and the red arrow indicates inflammatory cell infiltration. (b) Representative histological photos of the small intestinal villus in each group (×100). (c) Histological injury index of the colon. (d) The length of the small intestinal villus. Data are from 3 independent experiments, and all values are presented as the mean ± SEM. ^*∗*^*P* < 0.05 and ^*∗∗∗*^*P* < 0.001 compared to the untreated group; ^###^*P* < 0.001 compared to the DSS group.

HE staining indicated that DSS treatment induced severe inflammation, cell infiltration, and crypt loss (Figure 2(a)). The inflammatory response in the colons of mice in the CSWE groups and the 5-ASA group showed a distinct decrease in these changes compared with the colons of mice in the DSS-induced UC group. The columns in Figure 2(c) show the histopathological scores of each group. As shown in Figure 2(b), UC mice treated with CSWE or 5-ASA showed improvements in the damage to the small intestinal villi and significant restoration of the length of the small intestinal villi (Figures 2(c) and 2(d)), indicating the good therapeutic effect of CSWE and 5-ASA in UC mice.

### 3.3. Effects of CSWE on Proinflammatory Cytokines in the Colon of DSS-Induced UC Mice

**Figure 3:**
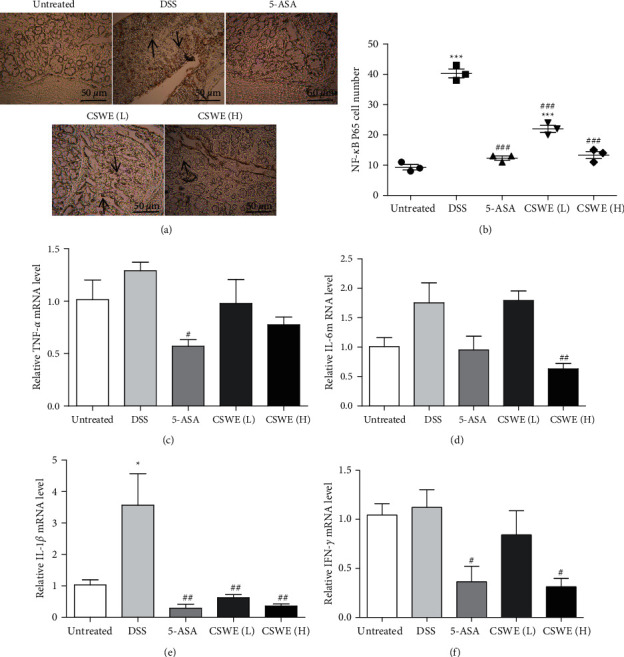
Effects of CSWE on proinflammatory cytokine expression in the colon of DSS-induced UC mice. (a) NF-*κ*B P65 immunohistochemical staining (×200). (b) NF-*κ*B P65 number of positive cells. Expression of inflammatory cytokine mRNA. (c) *TNF-α*. (d) *IL-1β*. (e) *IL*-6. (f) *IFN-γ*. Data are from 3 independent experiments, and all values are presented as the mean ± SEM. ^*∗*^*P* < 0.05 and ^*∗∗∗*^*P* < 0.001 compared to the untreated group, ^#^*P* < 0.05, ^##^*P* < 0.01, and ^###^*P* < 0.001 compared to the DSS group.

Immunohistochemical results showed that, compared with the control group, the number of NF-*κ*B P65-positive cells in the DSS group and CSWE(L) group increased significantly; however, the number of NF-*κ*B P65-positive cells in the CSWE(H) group and 5-ASA group remarkably decreased in comparison with that in the DSS group (Figures 3(a) and 3(b)). Next, qRT-PCR was used to examine the effect of CSWE on the release of proinflammatory cytokines in the colon. As shown in Figures 3(c)–3(f), compared with the untreated group, the expression of cytokines, including *TNF-α*, *IL-*1*β*, *IL-*6, and *IFN-γ*, was significantly increased in the DSS group (*P* < 0.01). Compared to the DSS group, the expression of these cytokines was reduced greatly in a dose-dependent manner in the groups administered CSWE.

### 3.4. CSWE Ameliorates DSS-Induced Epithelial Permeability by Enhancing the Function of the Gut Barrier

**Figure 4:**
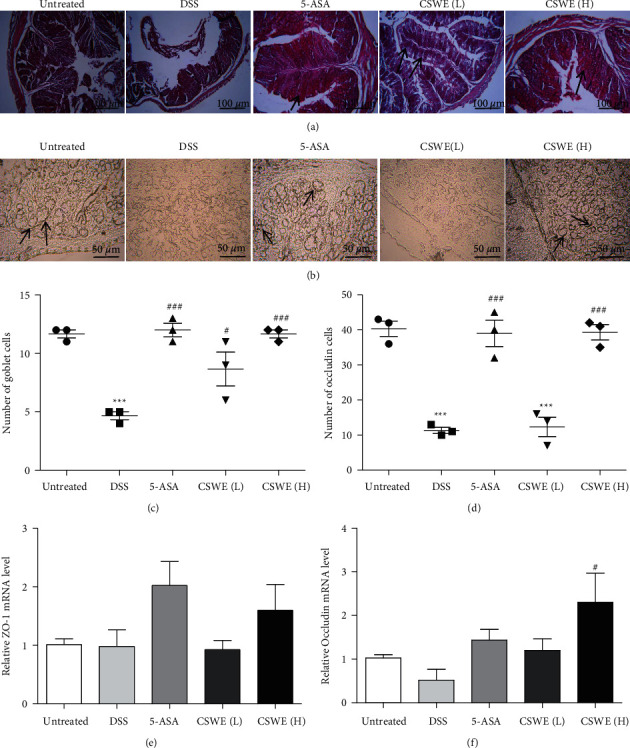
Effects of CSWE on the function of the mucus layer barrier in the colon of DSS-induced UC mice. (a) PAS staining of mucus in colonic sections (×100); the black arrow indicates goblet cells. (b) Occludin immunohistochemical staining (200×). (c) Number of goblet cells. (d) Number of occludin cells. (e) The relative expression levels of *ZO-*1 were detected by qRT-PCR. (f) The relative expression levels of *occludin* were detected by qRT-PCR. Data are from 3 independent experiments, and all values are presented as the mean ± SEM. ^*∗∗∗*^*P* < 0.001 compared to the untreated group; ^#^*P* < 0.05 and ^###^*P* < 0.001 compared to the DSS group.

Compared with untreated mice, serious depletion of mature goblet cells with fewer filled goblet cells and associated mucus were observed in DSS-treated mice by PAS staining (Figures 4(a) and 4(c)). Importantly, CSWE(H) and 5-ASA treatment remarkably improved the loss of mucus-producing goblet cells. Consistently, qRT-PCR results showed that CSWE treatment increased the levels of tight junction protein genes (*ZO-*1) and *occludin* in intestinal epithelial cells (Figures 4(e) and 4(f)). At the same time, the expression of *occludin* was also confirmed by immunohistochemical staining and quantification (Figures 4(b) and 4(d)).

### 3.5. CSWE Reduces Oxidative Stress in DSS-Induced UC Mice

**Figure 5:**
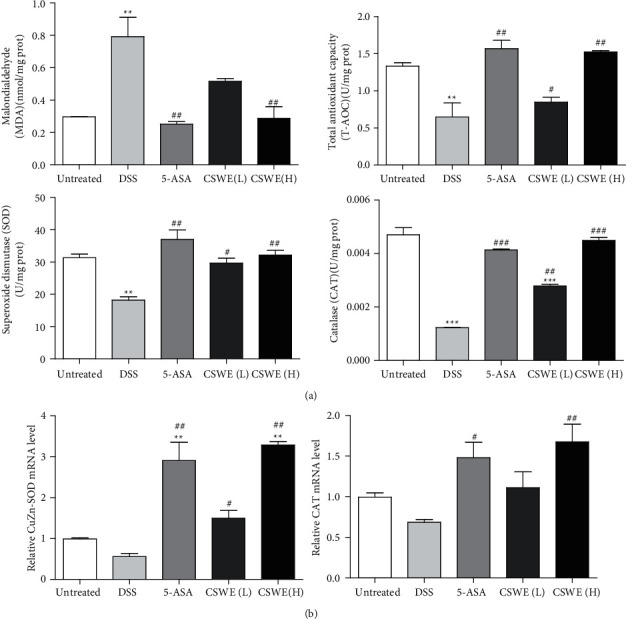
Effects of CSWE on oxidative stress in DSS-induced UC mice. (a) The MDA content, SOD and CAT activities, and T-AOC expression in colon tissue. (b) The relative expression levels of *CuZn-SOD* and *CAT* were detected by qRT-PCR. Data are from 3 independent experiments, and all values are presented as the mean ± SEM. ^*∗∗*^*P* < 0.01and ^*∗∗∗*^*P* < 0.001 compared to the untreated group;^#^*P* < 0.05, ^##^*P* < 0.01, and ^##^*P* < 0.001 compared to the DSS group.

The MDA content in colon tissue was markedly increased, whereas the SOD and CAT activities and T-AOC expression were significantly decreased in the DSS group (*P* < 0.01). However, significant improvement was observed after CSWE treatment in UC mice (Figure 5(a)). Furthermore, the expression patterns of some antioxidant enzymes, such as *CuZn-SOD* and *CAT*, were confirmed by qRT-PCR (Figure 5(b)). The expression of *CuZn-SOD* and *CAT* was downregulated in the DSS group. Conversely, CSWE enhanced the mRNA expression levels of *CuZn-SOD* and *CAT* in a dose-dependent manner in the colon compared with that in the DSS group.

### 3.6. CSWE Changes the Composition of Gut Microbes in DSS-Induced UC Mice

**Figure 6:**
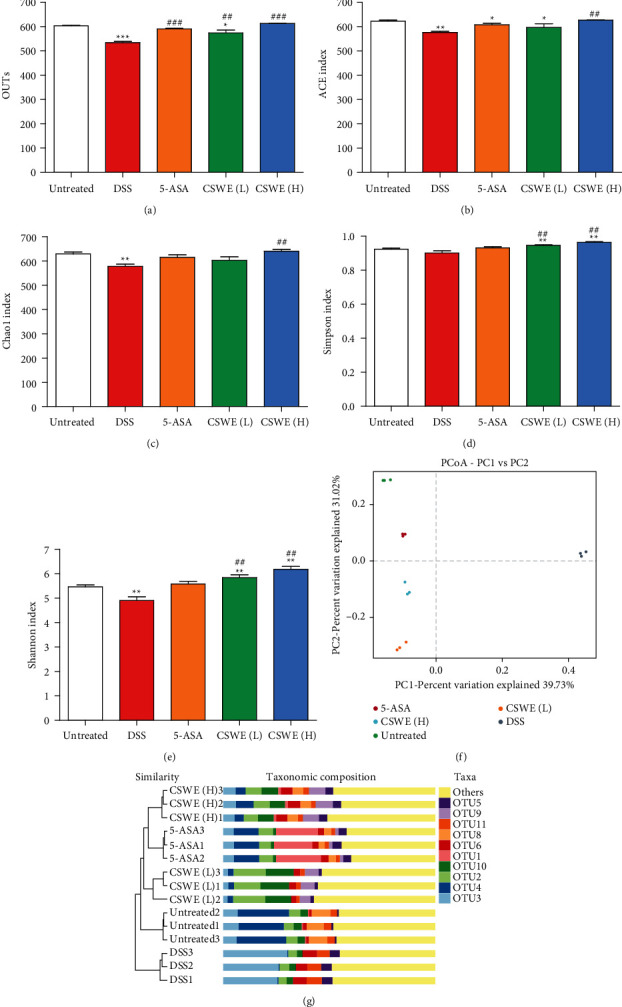
Effects of CSWE on the gut microbiota in DSS-induced UC mice. (a) OTU number for each group of samples. (b–e) Alpha diversity analysis diagram of the ACE index, Chao index, Simpson index, and Shannon index. (f-g) Beta diversity analysis of PCoA and hierarchical clustering. Data are from 3 independent experiments, and all values are presented as the mean ± SEM. ^*∗*^*P* < 0.05, ^*∗∗*^*P* < 0.01, and ^*∗∗∗*^*P* < 0.001 compared to the untreated group; ^##^*P* < 0.01 and ^###^*P* < 0.001 compared to the DSS group.

16S rRNA was used to investigate the changes in gut microbiota in UC mice after treatment with CSWE. The number of OTUs in the DSS group was extremely significantly decreased (*P* < 0.001) in comparison with that in the untreated group, which was improved by gavage with CSWE(H) and 5-ASA (Figure 6(a)). As shown in Figures 6(b)–6(e), the ACE, Chao, and Shannon indexes were extremely significantly reduced in the DSS group compared with the untreated group (*P* < 0.01); in contrast, the four indexes were significantly increased (*P* < 0.05) after CSWE treatment compared to the DSS group in a dose-dependent manner. Principal coordinates analysis (PCoA) and a hierarchical clustering tree were used to explore the similarities and differences in intestinal flora composition between different groups. A significant separation was observed between the DSS group and the other four groups (Figure 6(f)). The sample cluster tree is shown in Figure 6(g). The CSWE groups and 5-ASA group had similar gut microbial compositions, while the DSS group was separated from the other four groups.

**Figure 7:**
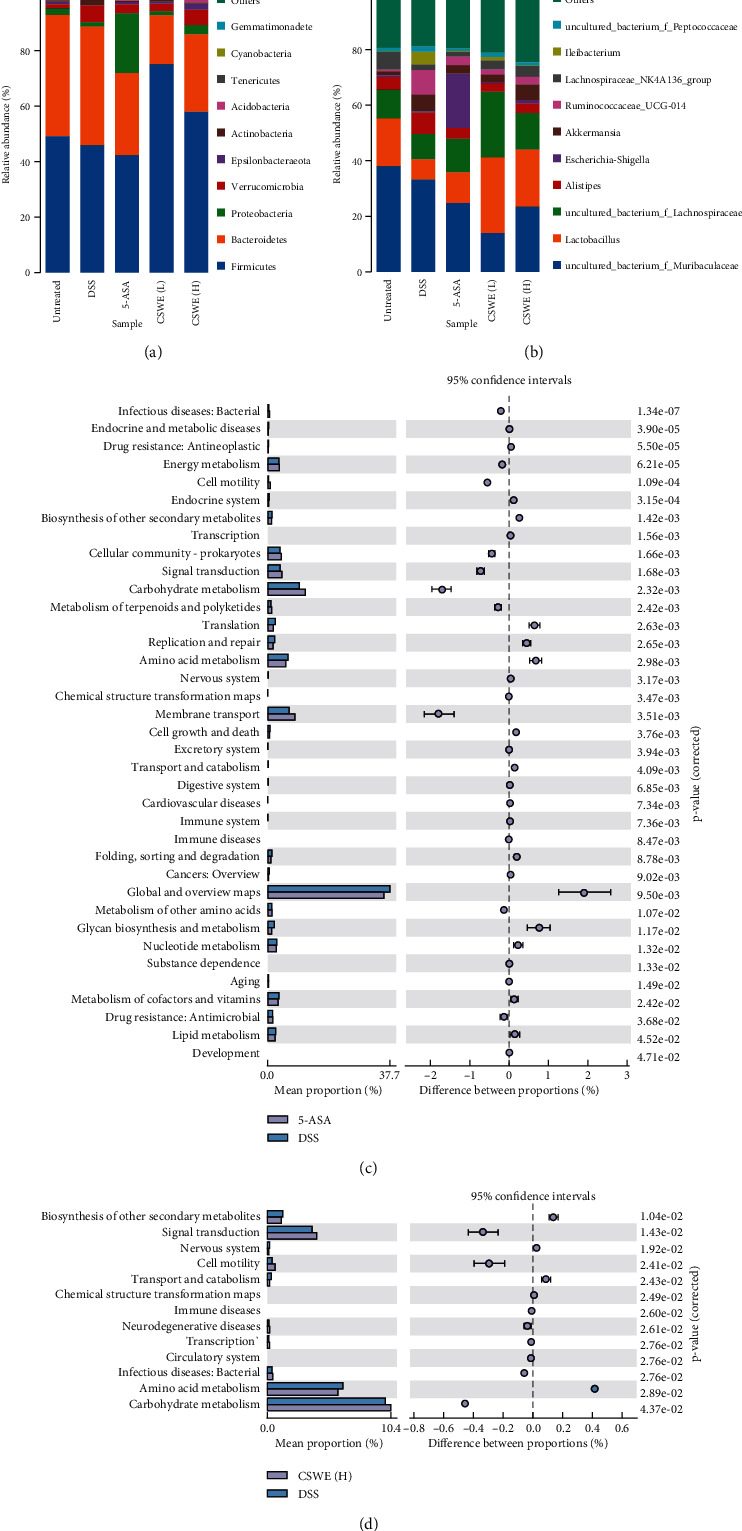
Gut microbial composition analysis and functional prediction. Gut microbial composition analysis at the phylum (a) and genus (b) levels. Gut microbial functional prediction. (c) Comparison between the DSS and 5-ASA group. (d) Comparison between the DSS and CSWE(H) groups.

Gut microbial composition analysis showed that CSWE changed the abundance of intestinal flora composition at different taxonomic levels. At the phylum level, the relative abundance of Firmicutes was significantly increased after CSWE treatment compared with the DSS group (*P* < 0.001), while Bacteroidetes and Actinobacteria were decreased (Figure 7(a)). At the genus level, the relative abundances of probiotics, including *Lactobacillus* and *Lachnospiraceae*, were increased significantly in the CSWE groups (*P* < 0.001) compared with the DSS group (Figure 7(b)). For expression of function prediction results of PICRUSt2, compared with the DSS group, the signal transduction and carbohydrate metabolism were obviously enhanced after CSWE(H) treatment, which was consistent with that of the 5-ASA group (Figures 7(c) and 7(d)). These results indicated that the gut microbial changes caused by DSS were alleviated after CSWE and 5-ASA treatment.

### 3.7. Fecal Metabolic Profiling and Its Correlation with the Gut Microbiota

**Figure 8:**
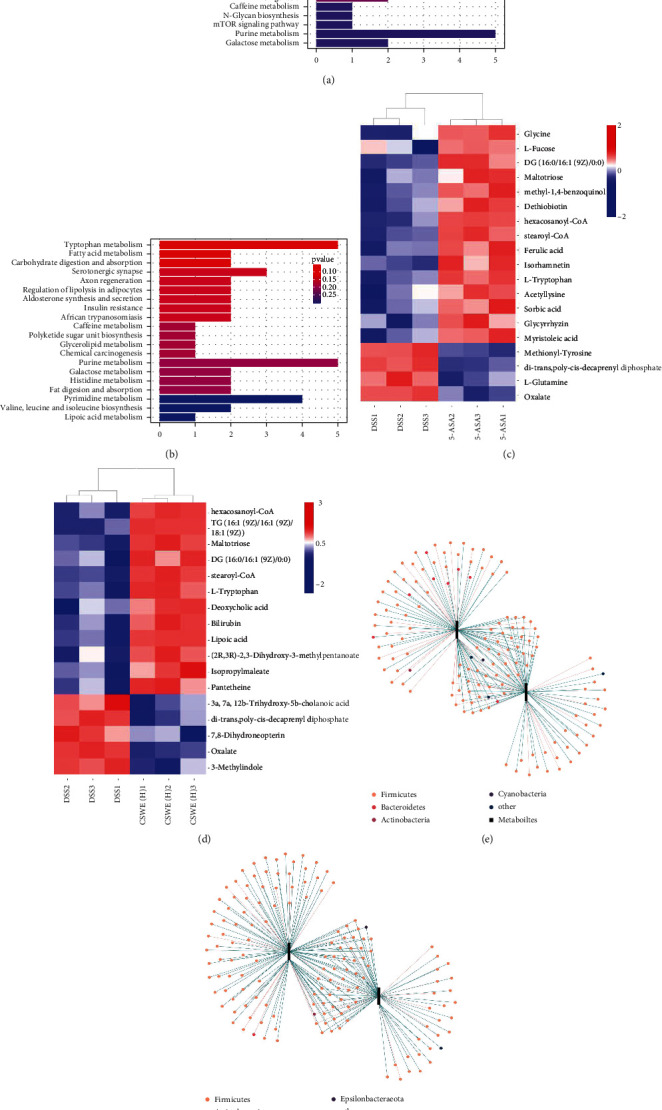
Effect of CSWE on the metabolism of gut microbiota in DSS-induced UC mice; enrichment analysis of KEGG metabolic pathways. (a) Comparison between the DSS and 5-ASA groups. (b) Comparison between the DSS and CSWE(H) groups. Heat map of metabolites with significant differences based on LC-MS data of fecal samples. (c) Comparison between the DSS and 5-ASA groups. (d) Comparison between the DSS and CSWE(H) groups. The relationships between metabolites and microorganisms. (e) Comparison between the DSS and 5-ASA groups. (f) Comparison between the DSS and CSWE(H) groups.

Enrichment analysis of KEGG metabolic pathways showed the top 20 different metabolic pathways between the 5-ASA and CSWE(H) groups compared with the DSS group (Figure 8(a)). Significantly, fatty acid metabolism, carbohydrate digestion, and absorption pathways are remarkably enhanced after CSWE(H) treatment. The heat map of HAC showed that 15 and 12 metabolites were significantly higher in the 5-ASA and CSWE(H) groups than in the DSS group, including L-tryptophan, isorhamnetin, and diacylglycerols (DG). In addition, both oxalate and di-trans poly-cis-decaprenyl diphosphate were significantly lower in the 5-ASA and CSWE(H) groups compared to the DSS group (Figure 8(b)). Global metabolomics and network analysis were performed to obtain the relationships between metabolites and microbes. As shown in Figure 8(c), the bacteria in Firmicutes were most closely related to metabolites.

## 4. Discussion

Considering the danger of UC to human health, it is essential to find alternative or supplementary therapeutic drugs for UC treatment. Drugs that are currently known to treat UC are associated with serious side effects. Therefore, safe and effective natural plant-derived medicines for the treatment of UC need to be developed [5]. A growing body of research has reported that the polysaccharides present in many traditional Chinese medicines, such as *Tremella fuciformis* polysaccharides [23] and *Atractylodes macrocephala* polysaccharides [19], play a crucial role in the treatment of UC. In our study, 37.6% polysaccharides and 3.5% protein were detected in CSWE. It has been reported that the water extract of *C. spinosa* may also contain flavonoids (quercetin) [24], indoles, phenolic sterols [18], glycosides, and saponins [21]. Here, we verified that CSWE (polysaccharides are the main component) treatment could alleviate DSS-induced UC via regulation of gut microbiota, oxidative stress, and inflammatory mediators in mice. Therefore, it is worth further isolating and identifying the effective constituents in CSWE for treating UC.

The characteristics of DSS-induced UC in mice are marked body weight loss, diarrhea, and severe bloody stool [5]. Here, we found that CSWE treatment mitigated body weight loss and significantly increased the DAI score during UC development induced by DSS. In addition, CSWE considerably alleviated colon shortening and ameliorated pathological damage to the colon. Consequently, the health conditions of UC mice were visibly improved. These results suggested that CSWE might provide a potential alternative to prevent the progression of UC.

Inflammatory cytokines play important roles in the occurrence and progression of colitis. Numerous studies have revealed that *IFN-γ* and *TNF-α* are signature cytokines and are critical for cell-mediated inflammation [25]. It has been reported that excessive *IL-*1*β* leads to an increase in intestinal permeability, which promotes the activation of dendritic cells and macrophages [26]. Several studies have illustrated that blocking *IL-*6 signal transduction in chronic intestinal inflammation leads to remarkable inhibition of colitis activity [27]. Here, we found that CSWE treatment could inhibit the expression of *TNF-α*, *IL-*1*β*, *IL-*6, and *IFN-γ*, which indicated that CSWE suppressed colonic inflammatory responses by decreasing the levels of proinflammatory cytokines in DSS-induced UC. Studies have shown that the cell-specific role of NF-*κ*B has been shown to be involved in the pathogenesis of IBD and that NF-*κ*B p65 is significantly enhanced in macrophages and epithelial cells isolated from inflammatory bowel specimens from IBD patients. Phosphorylation of I*κ*B*α*, an important inhibitor of NF-*κ*B signaling, leads to activation of NF-*κ*B signaling, allowing translocation of phosphorylated NF-*κ*B p65 into the nucleus, which regulates the production of inflammatory cytokines and chemokines such as TNF-*α*, IL-1, and IL-6, which directly contribute to mucosal tissue damage [28–30]. Our results showed that DSS stimulation significantly increased the number of nuclear NF-*κ*B P65 positive cells, and CSWE treatment significantly decreased the number of nuclear NF-*κ*B P65 positive cells. Thus, in the present study, CSWE might inhibit the nuclear translocation of NF-kB to exhibit anti-inflammatory effects.

Intestinal inflammation is associated with defective intestinal epithelial tight junction barriers, which are an important pathogenic factor contributing to the development of IBD [31]. Therefore, the regulation of tight junction proteins in the intestine is important for UC treatment. The dominant cell types of the intestinal epithelium constitute absorptive enterocytes and goblet cells. These goblet cells secrete the mucus layer that spreads on the intestinal epithelium [32]. In the present study, the PAS staining and quantitative results confirmed that CSWE treatment remarkably improved the loss of mucus-producing goblet cells. Additionally, the qRT-PCR and immunohistochemical results confirmed that CSWE could significantly increase the expression of tight junction proteins, including *ZO-*1 and *Occludin*. These results indicated that CSWE improves intestinal barrier function through mucosal and epithelial barriers.

Oxidative stress and lipid peroxidation could promote free radical chain reactions and impair the integrity of the intestinal mucosa barrier. During colitis, proinflammatory factors activate phagocytes, which infiltrate the mucosa and stimulate the production of reactive oxygen species (ROS), and excessive reactive oxygen species (ROS) levels contribute to the pathogenesis of the inflammatory ulcerative disease [22, 33–35]. MDA is a representative product of lipid peroxidation induced by ROS, which can cause crosslinking in lipids, proteins, and nucleic acids. Usually, our bodies have defense mechanisms, such as antioxidant enzyme systems, including SOD, CAT, and peroxidase (POD), to prevent tissue oxidative damage from ROS [5]. In this study, the MDA content was increased in the DSS group, and the enzymatic activity of antioxidants, such as SOD and CAT, and the T-AOC, was dramatically reduced, whereas CSWE treatment significantly mitigated oxidative stress in the colon of DSS-induced UC mice by decreasing the MDA content and improving antioxidant capacity, indicating that CSWE could ameliorate the oxidative stress induced by DSS.

The gut microbiota is composed of trillions of bacteria, which participate in numerous interactions with the host and play important roles in modulating the host's immune system through bacterial components [18]. Gut microbial dysfunction could cause increased permeability of intestinal epithelial cells, trigger the mucosal inflammatory response, and promote the development of UC. Therefore, regulation of the intestinal microbiota has been deemed a therapeutic strategy for UC patients [36]. The DSS-induced acute rodent colitis model is a reasonable way to understand the changes in the gut microbiota, similar to those found in human IBD [37]. Polysaccharides have been proven to have the ability to regulate intestinal microbial ecology. In the present study, we investigated the effect of CSWE on the gut microbiota of UC mice by 16S rRNA sequencing technology. The analysis of *α*-diversity demonstrated that CSWE could enhance microbial community diversity, which was illustrated by the improvement of the ACE, Chao, and Shannon indexes. The analysis of *β*-diversity, including PCoA and hierarchical clustering tree generation, revealed that the overall microbial community structure significantly changed after CSWE treatment compared to that in the DSS group, indicating that the effects of CSWE were achieved by regulating the composition of the gut microbiota.

Firmicutes play a vital role in the regulation of the immune response by inhibiting the invasion of opportunistic pathogens and preventing intestinal inflammation [38]. Subsequently, a more detailed taxonomic analysis of microbial community composition (phylum and genus level) was delivered. We found that the abundance of the phylum Firmicum in the CSWE groups increased significantly compared with that in the DSS group. Probiotics were reported to restore the function of the disturbed mucosal barrier, adjust the imbalance of intestinal microbiota, inhibit competition of potential pathogens, improve local and systemic immunity, and enhance intestinal barrier function [39]. In the present study, CSWE treatment greatly promoted the growth and recovery of several probiotics, including *Lactobacillus* and *Lachnospiraceae*, which proved the probiotic effect of CSWE. Based on the PICRUSt2 function prediction results, carbohydrate metabolism in 5-ASA and CSWE(H) treated mice was higher than that in the DSS group, which could probably explain why 5-ASA and CSWE treatment could restore energy supplementation by increasing microbiota-mediated glucometabolism. These results suggested that treatment with CSWE could regulate gut microbiota richness and diversity in UC mice and effectively maintain the homeostasis of intestinal microbiota by shifting the gut microbiota structure and reestablishing the microecological balance of UC mice.

In recent years, metabolomic studies of fecal samples have found that metabolic phenotypic changes are associated with gut microbiota changes in the development of diseases [40]. Through fecal analysis, we compared the metabolic pathways, metabolite types, and the relationship between the microbial community and metabolites between the 5-ASA and DSS groups and the CSWE(H) and DSS groups. It has been reported that fatty acid metabolism genes are highly correlated with immune cell metabolism and that these genes may be potential targets for UC therapy [41]. Carbohydrate metabolism is the main source of metabolic energy for *Lactobacillus* and contributes to the ecological adaptability of *Lactobacillus* [42]. The metabolites of the top 20 different metabolic pathways between 5-ASA and CSWE(H) are mainly involved in the amino acid synthesis, enzyme synthesis, glucose metabolism, and fatty acid biosynthesis. Tryptophan metabolites are recognized to function as endogenous ligands for aryl hydrocarbon receptors, which are critical regulators of inflammation and immunity [43]. DG is involved in the activation of protein kinase C in many cell types, and upregulation of DG may suggest modification of the PKC pathway, which plays a crucial role in many aspects of the stability of the gastrointestinal environment and is involved in many physiological and pathological processes, such as development, inflammation, and tumorigenesis [44]. In addition, DG can inhibit the secretion of bile acids to prevent diarrhea and the occurrence of colitis [45]. The results of the heatmap of HAC show that L-tryptophan and DG were significantly higher in both the 5-ASA and CSWE(H) groups. In our results, compared with DSS treatment, isorhamnetin was significantly higher in both the 5-ASA and CSWE(H) groups, which was consistent with the downregulation of *TNF-α*. Overaccumulation of oxalate leads to oxalate toxicity, which has multiple pathogenic manifestations [46]. We found that 5-ASA and CSWE(H) treatment decreased the oxalate content, which may be related to the degradation of oxalate by the gut microbiota. In addition, the results of metabonomics and microbial association analysis showed that metabonomics had the strongest association with Firmicutes in microorganisms. These results suggest that CSWE can improve DSS-induced UC by regulating gut microorganisms and metabolomics.

In conclusion, CSWE exhibited a protective effect in DSS-induced UC mice through inhibiting the release of proinflammatory cytokines, increasing tight junction proteins, improving antioxidant activity, and modulating the gut microbiota and regulatory metabonomics. Our results indicated that CSWE might be an ideal candidate as a potential therapeutic natural product for the treatment of UC. However, the bioactive components of CSWE and its mechanism in the treatment of UC need to be further purified and investigated.

## Figures and Tables

**Table 1:** Evaluation of the histological pathological score.

Score	Epithelial cells	Inflammatory cell infiltration
0	Normal form	No infiltration
1	Goblet cell loss	Infiltration in the basal layer of the crypt
2	Large area loss of goblet cells	Infiltration reaches the mucosal muscle layer
3	Crypt cell loss	Infiltration deep into the mucosal muscle layer, accompanied by mucosal thickening and edema
4	Large area loss of crypt cells	Infiltration to the submucosa

**Table 2:** Sequences of primers used for qRT-PCR.

Gene	Forward sequence	Reverse sequence
*TNF-α*	CCGATGGGTTGTACCTTGTC	GTGGGTGAGGAGCACGTAGT
*IL-*6	GACAACCACGGCCTTCCCTA	GGTACTCCAGAAGACCAGAGGA
*IL-*1*β*	GCAACTGTTCCTGAACTCAACT	ATCTTTTGGGGTCCGTCAACT
*IFN-γ*	ACACTGCATCTTGGCTTTGC	CCAGTTCCTCCAGATATCCA
*ZO-*1	AGAAGATAGCCCTGCAGC	AGTCCGTAAGGAGATTCT
*Occludin*	GGTCAGGGAATATCCACC	ATTATATTCATCAGCAGC
*CuZn-SOD*	AAGGCCGTGTGCGTGCTGAA	CAGGTCTCCAACATGCCTCT
*CAT*	GCAGATACCTGTGAACTGTC	GTAGAATGTCCGCACCTGAG
*GAPDH*	AGCCTCGTCCCGTAGACA	CTCGCTCCTGGAAGATGG

## Data Availability

All data included in this study are available upon request to the corresponding author.

## Abbreviations

5-ASA: Mesalazine
CAT: Catalase
CSWE: *Capparis spinosa* water extract
CuZn-SOD: CuZn-superoxide dismutase
DG: Diacylglycerol
DSS: Dextran sulfate sodium
*GAPDH*: Glyceraldehyde-3-phosphate dehydrogenase
HAC: Hierarchical clustering
IBD: Inflammatory bowel disease
*IFN-γ*: Interferon-*γ*
*IL-*1*β*: Interleukin-1*β*
*IL-*6: Interleukin-6
MDA: Malondialdehyde
SOD: Superoxide dismutase
T-AOC: Total antioxidant capacity
*TNF-α*: Tumor necrosis factor-*α*
UC: Ulcerative colitis
*ZO-*1: Zonula occludens-1.
